# Optimizing Irrigation and Nitrogen Application to Enhance Millet Yield, Improve Water and Nitrogen Use Efficiency and Reduce Inorganic Nitrogen Accumulation in Northeast China

**DOI:** 10.3390/plants13213067

**Published:** 2024-10-31

**Authors:** Tangzhe Nie, Jianfeng Li, Lili Jiang, Zhongxue Zhang, Peng Chen, Tiecheng Li, Changlei Dai, Zhongyi Sun, Shuai Yin, Mengxue Wang

**Affiliations:** 1 School of Water Conservancy and Electric Power, Heilongjiang University, Harbin 150080, China; 2019036@hlju.edu.cn (T.N.); 2222014@s.hlju.edu.cn (J.L.);; 2 Key Laboratory of Effective Utilization of Agricultural Water Resources, Ministry of Agriculture and Rural Affairs, Northeast Agricultural University, Harbin 150030, China; 3 School of Water Conservancy and Civil Engineering, Northeast Agricultural University, Harbin 150030, China; 4 College of Agricultural Science and Engineering, Hohai University, Nanjing 211100, China; 5 Institute of Geographic Sciences and Natural Resources Research, Chinese Academy of Sciences, Beijing 100101, China; 6 College of Ecology and Environment, Hainan University, Haikou 570208, China; 7 State Key Laboratory of Remote Sensing Science, Aerospace Information Research Institute, Chinese Academy of Sciences, Beijing 100101, China; 8 College of Agriculture, Heilongjiang Bayi Agricultural University, Daqing 163319, China

**Keywords:** millet, yield, water use efficiency, nitrogen agronomic efficiency, nitrogen accumulation, water and nitrogen optimization

## Abstract

Enhancing irrigation and nitrogen fertilizer application has become a vital strategy for ensuring food security in the face of population growth and resource scarcity. A 2-year experiment was conducted to determine to investigate the effects of different irrigation lower limits and nitrogen fertilizer application amounts on millet growth, yield, water use efficiency (WUE), N utilization, and inorganic nitrogen accumulation in the soil in 2021 and 2022. The experiment was designed with four irrigation lower limits, corresponding to 50%, 60%, 70%, and 80% of the field capacity (FC), referred to as I_50_, I_60_, I_70_, and I_80_. Four nitrogen fertilizer application were also included: 0, 50, 100, and 150 kg·hm^−2^ (designated as F_00_, F_50_, F_100_, and F_150_), resulting in a total of 16 treatments. Binary quadratic regression equations were established to optimize the irrigation and nitrogen application. The results demonstrated that the plant height, stem diameter, leaf area index, aboveground biomass, yield, spike diameter, spike length, spike weight, WUE, and nitrogen agronomic efficiency for millet initially increased before subsequently decreasing as the irrigation lower limit and nitrogen fertilizer application increased. Their maximum values were observed in the I_70_F_100_. However, the nitrogen partial factor productivity (PFPN) exhibited a gradual decline with increasing nitrogen application, reaching its peak at F_50_. Additionally, PFPN displayed a pattern of initial increase followed by a decrease with rising irrigation lower limits. The accumulation of NO_3_^−^-N and NH_4_^+^-N in the 0~60 cm soil layer increased with the increase of nitrogen fertilizer application in both years, while they tended to decrease as the irrigation lower limit increased. An optimal irrigation lower limit of 64% FC to 74% FC and nitrogen fertilizer application of 80 to 100 kg ha^−1^ was recommended for millet based on the regression equation. The findings of this study offer a theoretical foundation and technical guidance for developing a drip irrigation and fertilizer application for millet cultivation in Northeast China.

## 1. Introduction

Food security has emerged as one of the major challenges confronting the world today, driven by a dramatic increase in food demand due to the continuous expansion of the population and sustained increase in consumption levels [1,2]. Meanwhile, global agriculture is facing the dual challenges of declining production growth and rising environmental costs [3]. As the fourth most widely cultivated food crop globally, millet’s high-yielding nature play an important role in addressing the substantial gap between the production growth of staple crops like rice and maize and the increasing global population [4,5,6,7,8]. The implementation of scientific management and the efficient use of water and nitrogen are becoming pivotal factors in achieving high millet yield targets [9]. Improving water use efficiency has emerged as the key objective for raising crop yields in light of the severe worldwide scarcity of water resources [10,11,12]. Additionally, agricultural nitrogen inputs also have to cope with the issue of high costs and relatively low yields [13,14]. Excessive application of nitrogen fertilizer influences crop absorption as well as nitrogen loss, causing instability in yield [15,16,17]. Moreover, nitrogen overapplication also exacerbates environmental pollution [18,19].

Currently, a great proportion of millet is cultivated using rainfall as the primary water source; however, the implementation of effective irrigation strategies remains essential for maintaining millet yield [20,21]. Research by Matsuura et al. [22] and Jahansuz et al. [23] indicated that millet yields might decline due to insufficient water supply. Additionally, changes in irrigation quota have also impacted crop water use efficiency (WUE). For instance, Zhang et al. [24] and Nagaz et al. [25] demonstrated that reduced irrigation quota significantly increased WUE of millet. Seghatoleslami et al. [26] indicated that while WUE improved under diminished irrigation, millet yields significantly declined. Existing drip irrigation technologies have significantly improved the soil moisture environment by regulating moisture evaporation and infiltration [27,28]. This enhancement facilitated better water uptake and use by crops [29,30]. Ensuring high grain yields while enhancing WUE through drip irrigation technology has become crucial. Additionally, identifying an optimal irrigation interval within range of 50% to 80% of saturated soil moisture content represented an effective strategy for minimizing agricultural water use and promoting millet production [31].

Increased nitrogen application significantly enhanced millet yield [32,33]. Wenqian et al. [34] found that applying nitrogen fertilizer during the jointing stage led to significant improvement in nitrogen partial factor productivity (PFPN), nitrogen agronomic efficiency (AEN), and millet yield. However, it is noteworthy that the effective use of applied nitrogen by crops in agricultural production often exceeds less than half of the nitrogen input [35]. Rosolem [36] noted that increasing nitrogen fertilizer inputs led to elevated levels of NO_3_^−^-N and NH_4_^+^-N in the soil profile. The excessive nitrogen in the soil would be lost through processes such as nitrification and volatilization, which not only reduced the crop’s absorption and transformation efficiency of nitrogen fertilizer, but also deteriorated the soil structure [37,38,39]. The integration of drip irrigation with precise nitrogen application has been shown to substantially improve crop yields while effectively minimizing the depth and volume of inorganic nitrogen (including NO_3_^−^-N and NH_4_^+^-N) leaching [40]. Consequently, the management of soil NO_3_^−^-N and NH_4_^+^-N, alongside the optimization of crop yield, PFPN, and AEN, has emerged as a critical component for developing effective nitrogen application strategies in agriculture [41,42].

The response surface model (RSM) is a statistical method for solving multivariate problems [43]. This method allows for the optimization of multiple impact factors on the response variable by establishing relationships among these factors with a minimal number of experiments. By optimizing the strategies for irrigation and nitrogen in agriculture, RSM is able to achieve a joint maximum value for grain yield, as well as for water and nitrogen use efficiency, with a 95% confidence interval. For example, Zou et al. [44] optimized the water and fertilizer management system by fitting a quadratic equation, achieving 95% of the maximum values for maize yield, WUE, and net return simultaneously. Similarly, Yan et al. [45] employed RSM to identify the optimal range of irrigation and fertilizer application amounts, aiming to maintain high maize yields while minimizing NO_3_^−^-N in the soil profile. Furthermore, RSM has been extensively utilized to improve agricultural conditions for a diverse range of crops [46].

China ranks as the sixth largest producer of millet globally, with significant potential for further production expansion [47]. However, accelerated industrialization and urbanization in China have led to a continuous reduction in available land for grain cultivation. Consequently, increasing millet yields has become a crucial strategy to address the challenges of food security [48]. Heilongjiang Province plays a vital role in millet production in China [49]. Heilongjiang Province experiences significant seasonal and regional variations in rainfall patterns, highlighting the critical importance of irrigation in satisfying the water requirements of crops and achieving high yields [50]. Additionally, Heilongjiang Province is characterized by its rich black soil resources, renowned for their high productivity [51]. Maintaining soil health and ensuring the sustainable utilization of these resources are crucial for the advancement of regional agricultural development.

The objectives of this study were: (1) to determine the effects of different irrigation lower limits and nitrogen fertilizer application amounts on millet growth, yield, and its components, water and nitrogen use efficiency, and the accumulation of NO_3_^−^-N and NH_4_^+^-N in the soil profile through a 2-year experiment, and (2) to optimize irrigation and nitrogen application strategies by considering soil inorganic nitrogen accumulation, millet yield, and water and nitrogen use efficiency, aim at enhancing millet yield and improving the use of water and nitrogen resources. The findings of the study will provide a strong theoretical foundation and technical support for the implementation and widespread adoption of drip irrigation and fertilizer application systems for millet in Northeast China.

## 2. Results

### 2.1. Growth Indicators

**Figure 1:**
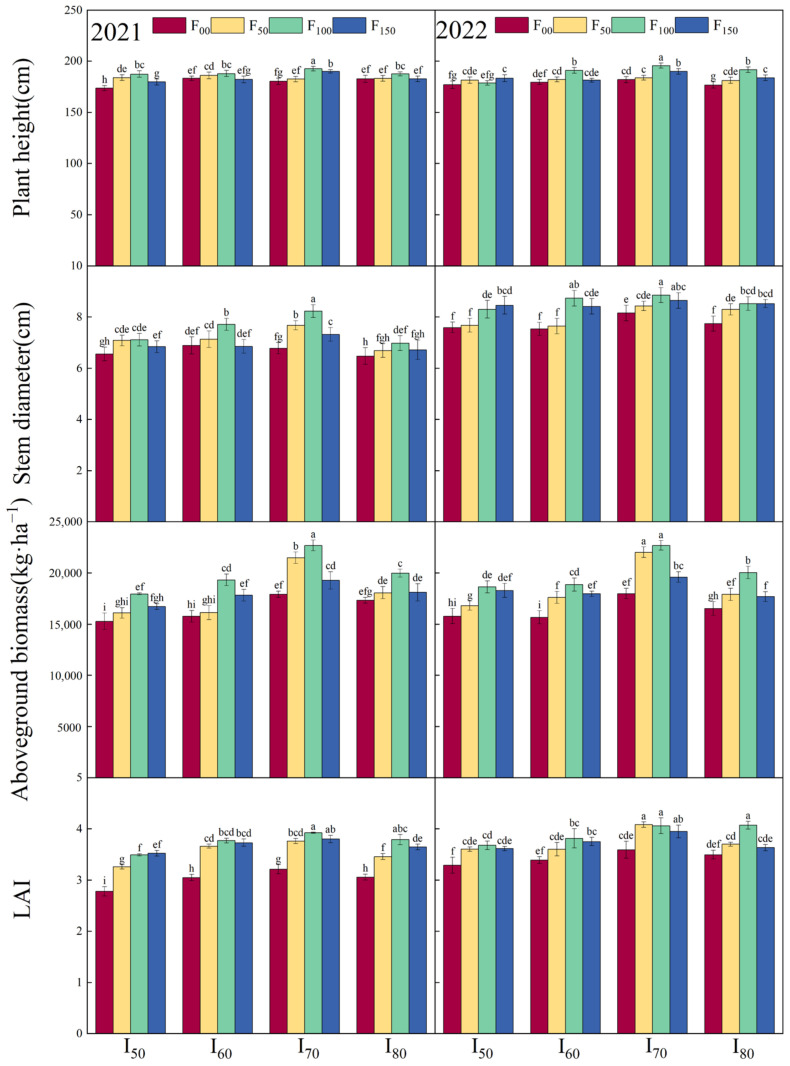
Plant height, stem diameter, leaf area index (LAI), and aboveground biomass of millet under different treatments in 2021 and 2022. I_50_, I_60_, I_70_, and I_80_ represent the irrigation lower limit being 50%, 60%, 70%, and 80% of the field capacity, respectively. F_00_, F_50_, F_100_, and F_150_ represent the nitrogen application amount of 0, 50, 100, and 150 kg·hm^−2^, respectively. Error bars represent one standard deviation about the mean. The letters above the bars are the mean separation indicators (LSD0.05), where similar letters indicate no significant difference.

As shown in Table 1, the lower limits of irrigation and nitrogen application amount significantly affected (*p* < 0.01) plant height, stem diameter, leaf area index (LAI), and the aboveground biomass of millet in both 2021 and 2022. Additionally, the interaction between these two factors had highly significant effects (*p* < 0.01) on plant height, stem diameter, and aboveground biomass. The plant height, stem diameter, LAI, and aboveground biomass initially increased and then decreased, with the increase of irrigation lower limits and nitrogen application amount. Their maximum values were observed under the I_70_F_100_ treatment (Figure 1). In 2021, the aboveground biomass was significantly higher in the I_70_F_100_ compared to other treatments. In 2022, both the I_70_F_100_ and I_70_F_50_ showed significantly greater aboveground biomass than other treatments. Notably, the aboveground biomass in all I_70_ treatments exceeded those of other irrigation treatments by 13.24% to 21.75%. The aboveground biomass in the F_100_ treatments was significantly greater than those of other nitrogen application treatments, showing an increase of 10.29% to 26.21%. Additionally, plant height and stem diameter under the I_70_F_100_ were increased by 10.33% to 10.52% and 22.26% to 22.54% compared to the I_50_F_00_. The LAI also exhibited a notable increase of 23.48% to 31.05% under I_70_F_100_ compared to I_50_F_00_.

### 2.2. Yield and Its Components

**Figure 2:**
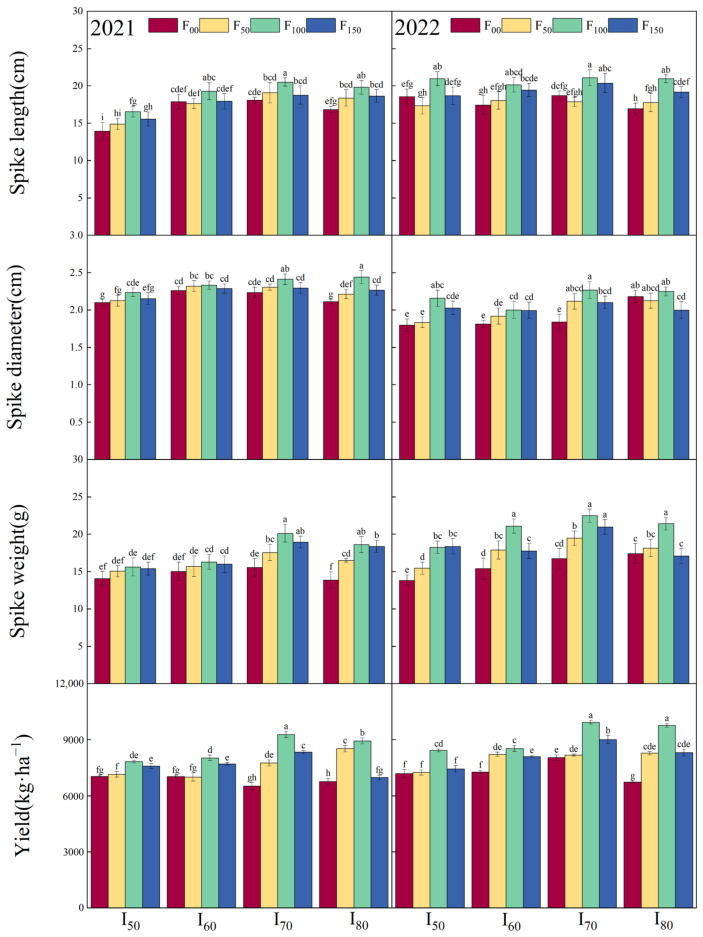
Spike diameter, spike length, spike weight, and yield of millet under different treatments in 2021 and 2022. I_50_, I_60_, I_70_, and I_80_ represent the irrigation lower limit being 50%, 60%, 70%, and 80% of the field capacity, respectively. F_00_, F_50_, F_100_, and F_150_ represent the nitrogen application amount of 0, 50, 100, and 150 kg·hm^−2^, respectively. Error bars represent one standard deviation about the mean. The letters above the bars are the mean separation indicators (LSD_0.05_), where similar letters indicate no significant difference.

As shown in Table 2, both the irrigation lower limit and nitrogen application amount had a highly significant effect (*p* < 0.01) on the spike length, spike stem, spike weight, and yield. The interaction between these two variables also had a significant effect (*p* < 0.01) on both the spike weight and yield. While the effect of irrigation lower limit and nitrogen application amount on the spike stem were significant (*p* < 0.05), no significant effect was found on the spike length. Overall, the spike length, spike diameter, spike weight, and yield increased with the increase of irrigation lower limit and nitrogen application amount, reaching maximum values under the I_70_ and F_100_ (Figure 2). The yields in F_100_ treatments were significantly higher than those in F_00_ and F_50_ treatments under I_50_ level in both years. In 2021, the yields of F_100_ were also significantly (*p* < 0.05) greater than those of other treatments under the I_60_ level. Over the two years, the F_100_ treatments consistently yielded significantly more than other treatments under the I_70_ and I_80_ levels. Additionally, in 2021, the yield of I_70_ was significantly (*p* < 0.05) greater than those of the other treatments when combined with F_100_ level. Furthermore, the yield of I_70_ was significantly greater than those of the I_60_ and I_50_ treatments when combined with the F_100_ level in 2022. The highest yield was observed in the I_70_F_150_ in both years. Additionally, the spike length, spike stem, spike weight, and yield were all significantly (*p* < 0.05) greater under I_70_F_100_ compared to the other treatments.

### 2.3. WUE, AEN and PFPN

**Figure 3:**
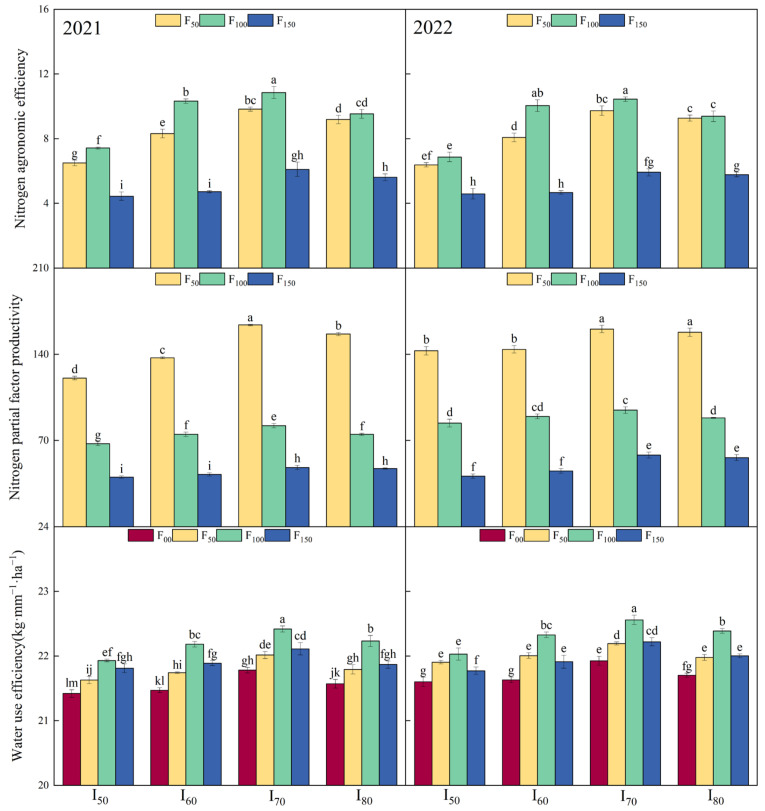
WUE, AEN, and PFPN of millet under different treatments in 2021 and 2022. I_50_, I_60_, I_70_, and I_80_ represent the irrigation lower limit being 50%, 60%, 70%, and 80% of the field capacity, respectively. F_00_, F_50_, F_100_, and F_150_ represent the nitrogen application amount of 0, 50, 100, and 150 kg·hm^−2^, respectively. Error bars represent one standard deviation about the mean. The letters above the bars are the mean separation indicators (LSD_0.05_), where similar letters indicate no significant difference.

As shown in Table 3, irrigation lower limit and nitrogen application amount significantly affected WUE, AEN, and PFPN (*p* < 0.01). The interaction between these two variables had a highly significant effect on both AEN and PFPN (*p* < 0.01). While this interaction did not significantly affect WUE in 2021, it did have a significant impact in 2022 (*p* < 0.05). Figure 3 illustrated that WUE initially increased then decreased with the increase of the irrigation lower limit and nitrogen application amount. In the F_100_ treatments, WUE was significantly higher under the I_70_ treatment in both 2021 and 2022. The WUE of I_70_ treatments increased by 7.51% to 15.53% compared to other irrigation treatments. Additionally, the WUE of the F_100_ treatment was significantly higher than those of the other nitrogen application treatments, with an increase of 10.12% to 27.33%. Both PFPN and AEN exhibited a trend of initially increasing and then decreasing in response to the irrigation lower limit. Specifically, PFPN displayed a downward trend with higher nitrogen application amount, peaking at the F_50_ level. In contrast, AEN showed an initial increase followed by a decline as nitrogen application increased, reaching its maximum value at the F_100_ level.

### 2.4. NO_3_^−^-N and NH_4_^+^-N Accumulation

**Figure 4:**
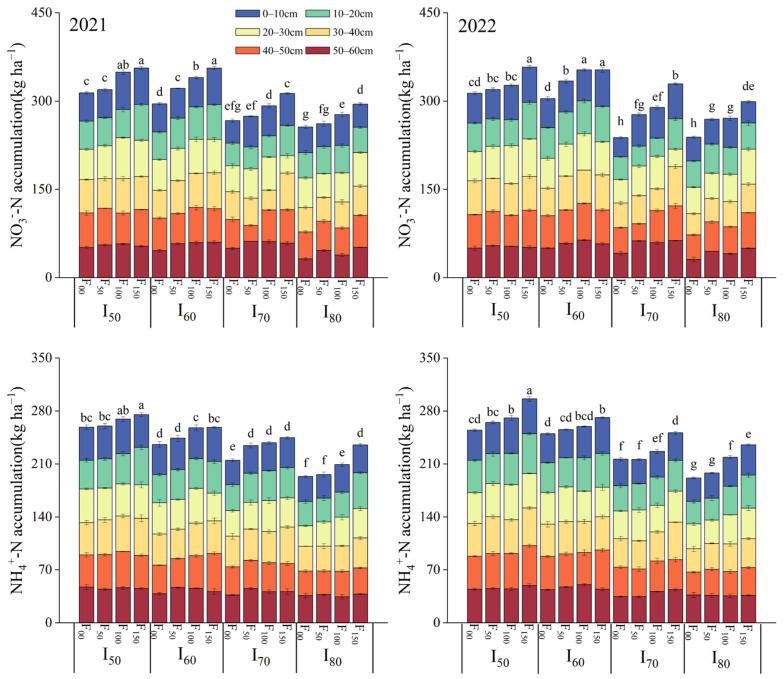
NO_3_^−^-N and NH_4_^+^-N accumulation in the 0~60 cm soil profile at harvest in the millet root zone in 2021 and 2022. I_50_, I_60_, I_70_, and I_80_ represent the irrigation lower limit being 50%, 60%, 70%, and 80% of the field capacity, respectively. F_00_, F_50_, F_100_, and F_150_ represent the nitrogen application amount of 0, 50, 100, and 150 kg·hm^−2^, respectively. Error bars represent one standard deviation about the mean. The letters above the bars are the mean separation indicators (LSD_0.05_), where similar letters indicate no significant difference.

As shown in Table 3, both the irrigation lower limit and nitrogen application amount significantly affected (*p* < 0.01) the accumulation of NO_3_^−^-N and NH_4_^+^-N in the soil. In 2021, the interaction between the irrigation lower limit and nitrogen application amount did not have a significant effect on NO_3_^−^-N accumulation. However, in 2022, this interaction was found to be highly significant (*p* < 0.01). Conversely, the interaction between the irrigation lower limit and nitrogen application amount had a highly significant (*p* < 0.01) or significant (*p* < 0.05) effect on NH_4_^+^-N accumulation in 2021 and 2022, respectively. The accumulation of NO_3_^−^-N and NH_4_^+^-N within the 0~60 cm soil layer showed a declining trend with increasing irrigation lower limit and an increasing trend with the increase of nitrogen application amount in both 2021 and 2022 (Figure 4). The percentages of NO_3_^−^-N and NH_4_^+^-N accumulation in the 0~10 cm, 10~20 cm, 20~30 cm, 30~40 cm, 40~50 cm, and 50~60 cm soil layers showed that the percentage of NH_4_^+^-N accumulation increased with soil depth, peaking at 17.44% in the 40~50 cm soil layer. However, NO_3_^−^-N accumulation was highest in the 50~60 cm soil layer, reaching 19.94%.

### 2.5. Optimization of Irrigation and Nitrogen Fertilizer

**Figure 5:**
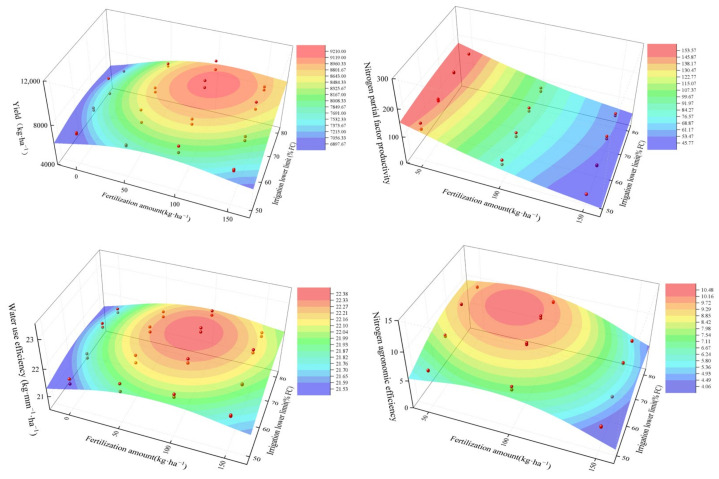
Relationship between yield, WUE, AEN, and PFPN under different irrigation lower limits and nitrogen fertilizer amounts. The red dots represent the measured values in 2021 and 2022. The green to red area represents the ≥95% confidence interval. FC: field capacity.

Binary quadratic regression equations were developed to optimize the irrigation and nitrogen fertilizer, using irrigation lower limits and nitrogen application as independent variables, while millet yield, WUE, AEN, and PFPN were designated as dependent variables (Table 4). The fitting results (Figure 5) indicated that each indicator achieved its optimal value under specified irrigation and nitrogen fertilizer conditions. Millet yield peaked at 9207.3 kg ha^−1^ at an irrigation lower limit of 73% field capacity (FC) and 108 kg ha^−1^ nitrogen fertilizer amount. The WUE peaked at 22.5 at 71% FC with 94 kg ha^−1^ of N. AEN peaked at 10.2 kg·ha^−1^·mm^−1^ at an irrigation lower limit of 74% FC and 79 kg ha^−1^ nitrogen fertilizer amount. Additionally, the PFPN reached a peak value of 158.2 at an irrigation lower limit of 79% FC and 50 kg ha^−1^ nitrogen fertilizer amount.

## 3. Discussion

### 3.1. Effects of Irrigation and Nitrogen Application on Millet Growth and Yield

The results of this experiment demonstrated a significant effect of nitrogen application amount on the aboveground biomass of millet (Table 1). The aboveground biomass continued to increase as the nitrogen application amount rose to F_100_ (Figure 1). This might be because the optimal nitrogen application amount stimulated photosynthesis, which subsequently has a beneficial impact on photosynthetic products and dry matter accumulation [32,52]. When the nitrogen application amount continued to rise until F_100_, the photosynthetic products contributed to higher yields (Figure 1). Gong et al. [53] noted that enhanced nitrogen supply promoted nutrient synthesis in cereal grains, leading to increased yields. In the current study, the observed increasing trend in spike length, spike diameter, and spike weight with the increasing nitrogen application amount was consistent with the increasing trend of yield, indicating a positive correlation between millet growth with yield (Figure 4) [54,55].

The present study demonstrated that irrigation had a highly significant impact (*p* < 0.01) on the yield and aboveground biomass (Table 1). Specifically, the I_70_ treatments resulted in a significantly higher yield compared to other irrigation treatments (Figure 1). This indicating that an irrigation lower limit of 70% FC created a more favorable soil water environment for millet growth, which facilitated better nitrogen absorption from the soil, ultimately leading to increased yield [25,56]. An increase in irrigation lower limit to I_80_ led to a reduction in yield, which likely due to excessive soil moisture content inhibiting root respiration of millet. Additionally, significantly lower yields were observed under I_50_ and I_60_ treatments, highlighting that insufficient irrigation also adversely affected millet yields [57]. The I_70_ treatments demonstrated a significantly higher aboveground biomass compared to other treatments (Figure 1), suggesting that optimal soil moisture conditions positively affect dry matter accumulation [58]. Moreover, the interaction between the irrigation lower limit and nitrogen application amount had a highly significant effect on grain yield (Table 2). By rationally regulating the ratio of these two factors, the efficiency of the nutrient uptake and translocation in grains can be significantly enhanced, leading to higher yields [5,59].

### 3.2. Effects of Irrigation and Nitrogen Application on Millet WUE, AEN and PFPN

The C4 crops exhibited greater resilience to water deficit conditions compared to other crop varieties. For instance, Seghatoleslami et al. [26] and Nagaz et al. [25] found that the WUE of millet improved when reducing irrigation. However, the results of this study showed that the I_70_ treatments demonstrated superior WUE (Figure 3). Zou et al. [44] indicated that increasing irrigation might be beneficial, as it allowed the crop to fully utilize available light and heat resources, ultimately making yield a primary determinant when calculating WUE. In this study, the WUE decreased as irrigation lower limit reduced, hitting a minimum in the I_50_ treatments. This decline could be attributed to the impact of decreased soil moisture on the crop’s ability to uptake and utilize soil nitrogen. Additionally, water deficit severely impaired grain growth and reduced yield, which in turn directly suppressed WUE [60,61,62,63]. The results of this study revealed that the I_80_ treatments had a significant inhibitory effect on the WUE of millet, suggesting that excessive water supply can be detrimental to grain WUE [64]. Additionally, the amount of nitrogen applied was found to significantly influence WUE in this study. The F_100_ treatments exhibited a higher level of WUE. This indicated that a moderate nitrogen fertilizer supply effectively mitigated the negative effects of water deficit on nitrogen uptake [65].

The results of this study demonstrated a decline in PFPN as the nitrogen application amount increased (Figure 3). Previous research by Lu et al. [16] indicated that higher levels of nitrogen application led to a sustained reduction in PFPN. Additionally, the irrigation lower limit significantly impacted PFPN, highlighting a notable interaction between irrigation and nitrogen application [66]. It was noteworthy that the PFPN of the I_70_ treatments were relatively higher than other treatments. This could be attributed to the favorable soil moisture conditions that improved the effectiveness of the applied nitrogen fertilizer [34,67]. In contrast, the AEN displayed a different pattern, initially increasing then declining as the nitrogen fertilizer application increased (Figure 3) Although the F_50_ treatment produced yields nearly close to those of the F_150_ treatments, their AEN were significantly higher, suggesting that high yields do not necessarily correlate with high AEN.

### 3.3. Effects of Irrigation and Nitrogen Application on NO_3_^−^-N and NH_4_^+^-N Accumulation

The dynamics of inorganic nitrogen in soil were affected by both irrigation and nitrogen management [68,69]. The application of nitrogen fertilizer significantly affected the accumulation of NO_3_^−^-N and NH_4_^+^-N within the 0–60 cm soil layer (Table 3). Furthermore, nitrogen application directly increased the accumulation of NO_3_^−^-N and NH_4_^+^-N in the soil (Figure 4). These findings were consistent with those of Giletto and Echeverría [70], who demonstrated that a higher nitrogen fertilizer amount led to a significant increase in the total accumulation of NO_3_^−^-N and NH_4_^+^-N in soil. Conversely, as the irrigation lower limit increased, the accumulation of NO_3_^−^-N and NH_4_^+^-N decreased. This trend could be attributed to the fact that increased soil moisture enhanced the vertical transport of inorganic nitrogen. Additionally, over-irrigation leads to the gravitational infiltration of NO_3_^−^-N and NH_4_^+^-N into deeper soil layers [45]. The current study revealed that the accumulation of NO_3_^−^-N and NH_4_^+^-N in the soil increased with soil depth. The highest accumulation of NO_3_^−^-N occurred at 50–60 cm, while NH_4_^+^-N peaked at 40–50 cm (Figure 4). This contrasted with the findings of Wang et al. [71], who reported a gradual decline in NO_3_^−^-N and NH_4_^+^-N accumulation with the increasing soil depth. This discrepancy might be due to the highly mobility of NO_3_^−^-N, which was more likely to infiltrate deeper soil layers, thereby leading to its accumulation in 50~60 cm soil layer [72,73]. In addition, NH_4_^+^-N was readily adsorbed by anionic colloids in the soil due to its positive charge; however, its mobility was relatively lower. Nonetheless, there was a tendency for NH_4_^+^-N to migrate to deeper soil layers, with peak accumulation often occurring at depths of 40–50 cm (Figure 4). In contrast, the accumulation of NO_3_^−^-N and NH_4_^+^-N in the shallow soil layer was relatively limited. This could be attributed to the combination of drip irrigation and fertilizer application methods, which enhanced inorganic nitrogen uptake by the grains and minimized nutrient loss. Although there were variations in the soil depths at which NO_3_^−^-N and NH_4_^+^-N were concentrated, both NO_3_^−^-N and NH_4_^+^-N were at risk of migrating to deeper soil layers. This further underscored the importance of nitrogen application and irrigation management. Despite this, our experimental data were analyzed to determine the optimal irrigation and nitrogen application corresponding to each response variable (Table 4). However, the combinations of irrigation lower limits and nitrogen applications associated with these optimal values were not identical. Consequently, this study aimed to identify the optimal irrigation lower limit and nitrogen application range within a 95% confidence interval (Figure 5). Additionally, the alterations in NO_3_^−^-N and NH_4_^+^-N in response to the irrigation lower limit and nitrogen application were evaluated (Figure 4). Based on the comprehensive analysis, this study recommended an optimal irrigation lower limit of 64% FC to 74% FC and a nitrogen fertilizer application of 80 to 100 kg ha^−1^.

This study presented the optimal ranges for irrigation and nitrogen application to support the sustainable production of millet in Northeast China, based on a two-year field experiment. To enhance the economic and ecological efficiency of agricultural production, future research should incorporate cost considerations related to irrigation and fertilization, providing a more comprehensive assessment from multiple perspectives, such as economic outcomes and yield. Furthermore, the use of isotope labeling techniques will enable more precise quantification of the specific impacts of nitrogen application on both yield and soil nitrogen accumulation, thereby establishing a solid foundation for informed scientific decision making [74].

## 4. Materials and Methods

### 4.1. Study Area

**Figure 6:**
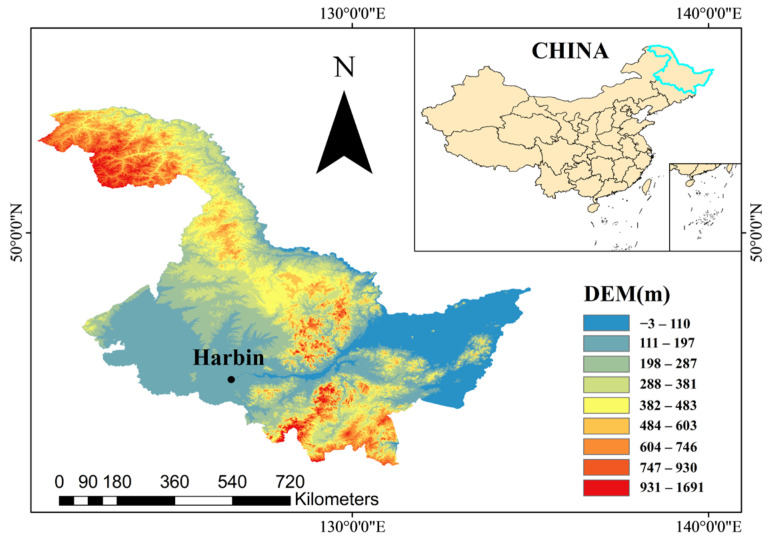
The geographical location of the study area.

**Figure 7:**
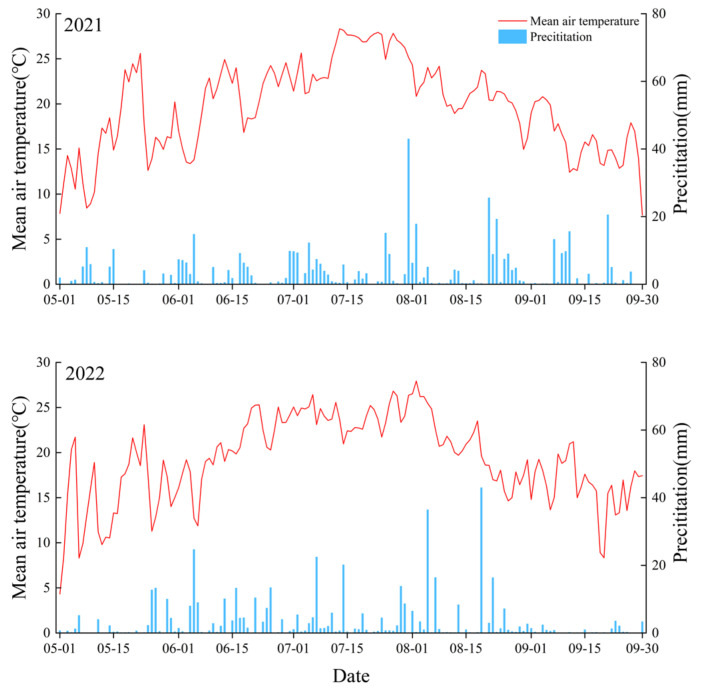
The mean air temperature and daily rainfall recorded at the study area during the millet growth periods in 2021 and 2022.

The study was conducted in the Xiangfang District (45°45′16.2″ N, 126°54′39.6″ E), Harbin, Heilongjiang Province, China (Figure 6). The study area experiences a cold–temperate monsoon climate, with precipitation unevenly distributed throughout the year, primarily occurring between July and September. During this period, cumulative precipitation accounts for approximately 70% of the total annual precipitation. The mean air temperature and precipitation during the millet growth period in 2021 and 2022 are presented in Figure 7. The basic experimental soil properties are shown in Table 5.

### 4.2. Experimental Design

The millet variety selected for this experiment was ‘Longgu 25’, which was sown on 23 May 2021 and 21 May 2022 and harvested on 5 September 2021 and 4 September 2022, respectively. Four lower limits of irrigation were established based on the FC: 50% FC (I_50_), 60% FC (I_60_), 70% FC (I_70_), and 80% FC (I_80_). The upper limit of irrigation was set at 100% FC for all treatments. Nitrogen application amount was divided into four levels: 0 kg·hm^−2^ (F_00_), 50 kg·hm^−2^ (F_50_), 100 kg·hm^−2^ (F_100_), and 150 kg·hm^−2^ (F_150_). The experiment comprised 16 treatments arranged in a completely randomized sequence with three replications, resulting in a total of 48 plots. Each experimental plot measured 60 m^2^ (10 m × 6 m). Fertilizer was applied in two stages: initially as a base fertilizer, followed by a single application of additional fertilizer. The initial application included 95 kg·ha^−1^ of P_2_O_5_, 55 kg·ha^−1^ of K_2_O and one-third of the nitrogen fertilizer, while the remaining two-thirds of the nitrogen fertilizer were applied at the end of the jointing stage of millet.

The experimental field was subdivided into 10 rows of ridges, with a distance of 60 cm between the ridges and 3.5 cm between the plants within each ridge. Drip irrigation tapes were installed in the middle of each ridge, by the roots of the millets, and both irrigation and nitrogen application were regulated using water meters and fertilizer tanks. Soil samples were collected from the center of the experimental field at 7-day intervals throughout the duration of the experiment using a soil auger and the five-point sampling method. Irrigation was initiated when the soil water content reached the lower limit of irrigation. To prevent interaction effects between different treatments, a 1.0 m buffer zone was established between each plot.

### 4.3. Sample Collection and Determination

#### 4.3.1. Growth Indicators and Yield Components of Millet

Plant samples were collected from five randomly selected plants for each plot. These plants were evenly distributed and well established at the maturity stage. The height of the samples was measured using a tape measure, while the stem diameter was measured using a vernier caliper. The total leaf area of the samples was measured using a YT-YMJ-02 Leaf Area Meter (Shandong Yuntang Intelligent Science and Technology Co., Shandong, China). After measuring the above parameters, the samples were placed in an oven at 105 °C for an initial drying period of 0.5 h. The oven temperature was then adjusted to 75 °C, and drying continued until a constant weight was achieved. Subsequently, the dry weight of each sample was measured using an electronic balance to calculate the aboveground biomass (kg·ha^−1^).

For each treatment, one square meter of grain ears was randomly selected for collection, and the following variables were measured: spike length, spike diameter, and spike weight. After threshing, the grains were air-dried and weighed, and the final yield was converted to grain yield at 14% moisture content. The yield was then calculated in kg·ha^−1^.

Leaf area index (*LAI*) was calculated as:

(1) $$LAI=\frac{Leaf\;area\;per\;plant}{Land\;area\;per\;plant}$$

#### 4.3.2. WUE

Irrigation (I) was calculated as [71]:

(2) $$I=10\times D\times H\times (W_{i}-W_{j})$$

where *I* is the irrigation volume of each treatment (kg m^−2^), *D* is the soil bulk density (g cm^−3^), *H* is the thickness of the soil layer (cm), *W_i_* is the target moisture content (%) of each soil layer from 0 to 60 cm, and *W_j_* is the current moisture content (%) of each soil layer from 0 to 60 cm.

Evapotranspiration (*ET*) was calculated as:

(3) $$ET=P+I+C_{r}-R_{o}-D_{w}-∆W$$

where *ET* is the crop evapotranspiration (mm), *P* is rainfall during the millet growth period (mm), Δ*W* is the change in soil moisture from the beginning to the end of the experiment (mm), *C_r_* is groundwater recharge (mm), *R_o_* is surface runoff (mm), and *D_w_* is deep drainage (mm). In this experiment, the effects of *C_r_*, *R_o_*, and *D_w_* were not considered [45].

WUE was calculated as [75]:

(4) $$WUE=\frac{Y}{ET}$$

where WUE is defined as water use efficiency (kg·ha^−1^·mm^−1^); *Y* represents the yield (kg·ha^−1^).

#### 4.3.3. AEN and PFPN

AEN was calculated as [76]:

(5) $$AEN=\frac{Y_{N}-Y_{C}}{N_{app}}$$

where *Y_N_* and *Y_C_* refer to the grain yields (kg·ha^−1^) of nitrogen and no nitrogen treatments, respectively. Finally, *N_app_* is the total nitrogen applied to each treatment (kg·ha^−1^).

PFPN was calculated as [77]:

(6) $$PFPN=\frac{Y_{N}}{N_{app}}$$

#### 4.3.4. NH_4_^+^-N and NO_3_^−^-N Accumulation in Soil

Soil samples were obtained from depths of 0~10 cm, 10~20 cm, 20~30 cm, 30~40 cm, 40~50 cm, and 50~60 cm, employing the identical sampling methodology utilized for the determination of soil water content. The soil samples were stored at −20 °C and subsequently analyzed for nitrogen content using an UV spectrophotometer (Hach DR5000). The accumulation of NO_3_^−^-N (*N_n_*) and NH_4_^+^-N (*N_a_*) was calculated as [78]:

(7) $$N_{n}orN_{a}=0.1\times C\times H\times D$$

where *N_n_* and *N_a_* represent soil NO_3_^−^-N and NH_4_^+^-N accumulations (kg·ha^−1^), respectively. *C* and *H* denote the soil NO_3_^−^-N or NH_4_^+^-N content (mg·kg^−1^) and the thickness of the soil layer (cm), respectively. *D* is the soil bulk density (g·cm^−3^).

### 4.4. Date Analysis

SPSS 18.0 (IBM Corp., New York, NY, USA) was employed for analysis of variance (ANOVA) and testing the significance between treatments. Figures were produced using Origin 2023b (Origin Lab Corporation, Northampton, MA, USA). A RSM analysis was conducted to visualize the effects of irrigation lower limit and nitrogen application amount on yield, WUE, AEN, and PFPN. Binary quadratic regression equations were developed by Origin 2023b (Origin Lab Corporation, Northampton, MA, USA). The objective was to identify the optimal regions for each response variable. This involved determining the combinations of irrigation lower limit and nitrogen application amount that would achieve the best response for each variable, as well as identifying ranges that would simultaneously satisfy the optimization requirements for all response variables. The optimization results were compared and overlapped to achieve this goal.

## 5. Conclusions

Millet yield, WUE, and AEN reached their maximum at the irrigation lower limit of 70% and 100 kg ha^−1^ nitrogen fertilizer amount, while the highest PFPN were found at the irrigation lower limit of 70% and 50 kg ha^−1^ nitrogen fertilizer amount. Within the 0~60 cm soil layer, as the nitrogen application rate increased, the accumulation of NH_4_^+^-N and NO_3_^−^-N showed an increasing trend. However, raising the irrigation lower limit helped reduce the NH_4_^+^-N and NO_3_^−^-N accumulation in soil. An optimal irrigation lower limit of 64% FC to 74% FC and a nitrogen fertilizer application of 80 to 100 kg ha^−1^ was recommended for enhancing millet yield, improving water and nitrogen use efficiency and reducing inorganic nitrogen accumulation in Northeast China. The agricultural economic and ecological effect worth further consideration when optimizing the irrigation and fertilizer management in future study.

## Figures and Tables

**Table 1:** ANOVA of irrigation lower limit and nitrogen fertilizer amount on plant height, stem diameter, LAI, and aboveground biomass of millet.

Factors	Plant Height		Stem Diameter		LAI		Aboveground Biomass	
	2021	2022	2021	2022	2021	2022	2021	2022
Irrigation lower limit	**	**	**	**	**	**	**	**
Nitrogen fertilizer amount	**	**	**	**	**	**	**	**
Irrigation lower limit × nitrogen fertilizer amount	**	**	**	**	ns	ns	**	**

Note: ** means a highly significant difference (*p* < 0.01); and ns means no significant difference (*p* > 0.05).

**Table 2:** ANOVA of irrigation lower limit and nitrogen fertilizer amount on spike diameter, spike length, spike weight, and yield of millet.

Factors	Spike Diameter		Spike Length		Spike Weight		Yield	
	2021	2022	2021	2022	2021	2022	2021	2022
Irrigation lower limit	**	**	**	ns	**	**	**	**
Nitrogen fertilizer amount	**	**	**	**	**	**	**	**
Irrigation lower limit × nitrogen fertilizer amount	*	**	ns	ns	**	**	**	**

Note: * means a significant difference (*p* < 0.05); ** means a highly significant difference (*p* < 0.01); and ns means no significant difference (*p* > 0.05).

**Table 3:** ANOVA of irrigation lower limit and nitrogen fertilizer amount on WUE, AEN, PFPN, NO_3_^−^-N accumulation, and NH_4_^+^-N accumulation.

Factors	WUE		AEN		PFPN		NO_3_^−^-N Accumulation		NH_4_^+^-N Accumulation	
	2021	2022	2021	2022	2021	2022	2021	2022	2021	2022
Irrigation lower limit	**	**	**	**	**	**	**	**	**	**
Nitrogen fertilizer amount	**	**	**	**	**	**	**	**	**	**
Irrigation lower limit × nitrogen fertilizer amount	ns	*	**	**	**	**	ns	**	**	*

Note: * means a significant difference (*p* < 0.05); ** means a highly significant difference (*p* < 0.01); and ns means no significant difference (*p* > 0.05).

**Table 4:** Regression equations for the response variables of yield, PFPN, AEN, WUE under different irrigation and nitrogen fertilizer conditions.

Response Variable	Regression Equations	R^2^
Yield	z = −3170.44 + 13.23 × x + 320.89 × y + 0.29 × x × y − 0.16 × x^2^ − 2.42 × y^2^	0.86
PFPN	z = 130.01 − 1.91 × x + 2.86 × y − 0.36 × 10^−3^ × x × y + 0.0046 x^2^ − 0.18 × 10^−1^ × y^2^	0.99
AEN	z = −28.44 + 0.19 × x + 0.85 × y − 0.55 × 10^−3^ × x × y−0.95 × 10^−3^ × x^2^−0.55 × 10^−2^ × y^2^	0.97
WUE	z = 17.77 + 9.29 × 10^−3^ × x + 0.12 × y + 0.42 × 10^−4^ × x × y−0.65 × 10^−4^ × x^2^ − 0.87 × 10^−3^ × y^2^	0.92

Note: x is the irrigation lower limit, and y is the nitrogen fertilizer amount.

**Table 5:** Soil properties.

Items	Value
Field capacity (%)	30.93
Total N content (g·kg^−1^)	0.27
Soil organic matter content (g·kg^−1^)	32.2
Total P content (g·kg^−1^)	1.07
Available phosphorus content (mg·kg^−1^)	24.81
Available potassium content (mg·kg^−1^)	262.22
pH	7.12
Soil texture	Loamy soil

## Data Availability

The raw data supporting the conclusions of this article will be made available by the authors on request.
